# Immunoglobulin M gene association with autoantibody reactivity and type 1 diabetes

**DOI:** 10.1007/s00251-017-0999-1

**Published:** 2017-05-22

**Authors:** Inês Rolim, Nádia Duarte, Gabriela Barata, João Costa, Luís Gardete-Correia, José Boavida, Rui Duarte, João Raposo, Zulmira Peerally, Manuela Catarino, Carlos Penha-Gonçalves

**Affiliations:** 1 Instituto Gulbenkian de Ciência, Apartado 14, P-2781-901 Oeiras, Portugal; 2 Faculdade de Farmácia, Universidade de Lisboa, Lisbon, Portugal; 3 Portuguese Diabetes Association, Education and Research Center, Lisbon, Portugal

**Keywords:** IgH locus, Type 1 diabetes, Autoantibodies, Anti-GAD, IgM, Genetic association

## Abstract

Several lines of evidence show that autoimmune responses evolving in type 1 diabetes (T1D) patients include the generation of multi-reactive autoantibody (AutoAb) repertoires, but their role in T1D pathogenesis remains elusive. We tested the hypothesis that variants at the immunoglobulin heavy chain (IGH) locus are genetic determinants of AutoAbs against pancreatic antigens and contribute to T1D susceptibility. With this aim, two independent study designs were used: a case-control study and a family-based cohort comprising a total of 240 T1D patients, 172 first-degree relatives (mother and/or father), and 130 unrelated healthy controls living in Portugal. We found that three SNPs in the IGH locus show suggestive association with T1D with the highest nominal association at rs1950942 (in the IGHM-IGHJ gene region) in both the case-control study (*P* = 9.35E−03) and the family-based cohort (*P* = 3.08E−03). These SNPs were also associated with IgG AutoAbs against pancreatic antigens and with AutoAb multi-reactivity in T1D patients. Notably, we found that the SNP with the highest association with T1D susceptibility and IgG autoantibody reactivity (rs1950942) was also associated with anti-GAD IgM reactivity in T1D patients (*P* = 5.98E−03) and in non-affected parents (*P* = 4.17E−03). This finding implies that IGH association with autoreactive IgM is detectable irrespective of disease status.

These results suggest that genetic variants at the IgM gene region of the IGH locus contribute to antibody autoreactivity and are associated with T1D. We propose that the control of autoantibody generation by *IGH* polymorphisms is a component of the complex architecture of T1D genetic susceptibility.

**Electronic supplementary material:**

The online version of this article (doi:10.1007/s00251-017-0999-1) contains supplementary material, which is available to authorized users.

## Introduction

Autoantibodies (AutoAbs) have proved to be pathogenic in several autoimmune diseases, but their role in type 1 diabetes (T1D) remains elusive (Notkins and Åke, 2001; Elliott et al. 2012). Autoimmune reactions against insulin-producing beta cells, in the pancreatic islets of Langerhans, are critical T1D pathogenic events where T cell-mediated activity is perceived to play a key role in the effector phase of beta cell destruction (Bach 1994; Roep and Peakman 2011). Nevertheless, B cells and the antibodies they secrete are not negligible. B cells were shown to contribute to beta cell autoimmunity in the NOD mouse model by presenting islet autoantigens to T cells or by secreting proinflammatory cytokines (Chan and Shlomchik 1998; Chan et al. 1999; Harris et al. 2000; Wong et al. 2004). In addition, B cell depletion in clinical trials showed beneficial effects in preserving beta cell function (Pescovitz et al. 2009). On the other hand, AutoAbs in T1D represent a clear manifestation that B cell tolerance has been disrupted (Ziegler et al. 1999; Kimpimäki et al. 2002; LaGasse et al. 2002). AutoAbs against islet antigens are detectable at very early pathogenesis stages and are instrumental indicators of preclinical diabetes progression (Yu et al. 2000). Anti-islet antibodies can enhance the expansion of islet-reactive CD4^+^ T cells and contribute to disease progression in a T1D transgenic mouse model (Harbers et al. 2007). Furthermore, anti-islet AutoAbs were shown to trigger murine autoimmune diabetes in the presence of an increased frequency of islet-reactive CD4 T cells (Silva et al. 2011).

AutoAb repertoires in T1D patients often display specificities typical of other autoimmunity diseases. Reportedly, T1D patients show high prevalence of concomitant autoimmune thyroid disease (15 to 30%) (Perros et al. 1995; Umpierrez et al. 2003), celiac disease (4–9%) (Bao et al. 1999; Carlsson et al. 1999; Aktay et al. 2001; Barera et al. 2002; Barker et al. 2005), or Addison’s disease (0.5%) (Barker et al. 2005). A large study demonstrated in T1D patients that occurrence of AutoAbs against antigens a priori not associated with diabetes is in part genetically controlled by non-HLA T1D susceptibility loci (Plagnol et al. 2011). These results reinforce the view that autoimmune responses evolving in T1D patients include the generation of multi-reactive AutoAb repertoires.

At large extent, the AutoAb research in T1D focuses on total IgG antibodies against anti-islet antigens, which result from adaptive immune responses. Nevertheless, high prevalence of IgG1 and IgG3 AutoAbs were associated with rapid progression to T1D (Hoppu et al. 2004a), indicating that different Ig isotypes may have distinct roles in disease. Although the IgM isotype is represented among the AutoAbs implicated in T1D, its association with disease pathogenesis is unclear (Dean et al. 1986; Hawa et al. 2000). It has been reported that the human fetus generates natural antibodies that recognize a uniform set of autoantigens, some of which are associated with autoimmune diseases (Merbl et al. 2007). Natural antibodies are produced in the absence of exogenous stimulation and are germline-encoded, low-affinity, polyreactive antibodies, mainly of the IgM isotype (Coutinho et al. 1995). This raises the possibility that faltering generation of natural and otherwise benign autoimmunity could result in autoimmune disease (Merbl et al. 2007). We have reported that the IgM repertoire of pre-insulitic NOD mice displays increased levels of B1 cell-derived anti-islet antibodies that are able to bind beta cells ex vivo and are generated irrespective of the NOD major histocompatibility haplotype (H-2) (Côrte-Real et al. 2012). Furthermore, it has been demonstrated that the murine repertoire of immunoglobulin heavy chain (IgH) gene rearrangements is dependent on the *IgH* haplotype (Viale et al. 1992) and is abnormal in the NOD mouse (Andersson et al. 1994).

We have noted that genome-wide SNP genotyping platforms show poor coverage of common frequency polymorphisms in the *IGH* locus (Supp. Table S1). This prompted us to investigate the role of the *IGH* locus in the genetic determination of AutoAb repertoires and in T1D susceptibility. Here, we tested the genetic association of *IGH* SNPs to T1D susceptibility and to antibody autoreactivity in two cohorts of Portuguese patients.

## Materials and methods

### Subjects and clinical criteria

This investigation was conducted under the ethical permission obtained for the study entitled “Estudo da base genética da imunopatologia associada à Diabetes Tipo 1 na população portuguesa” granted by the Ethics Committee of the Associação Protectora dos Diabéticos de Portugal (APDP) in Lisbon. Written, informed consent was obtained from the participants or parents of each child. All investigations have been conducted according to the principles expressed in the Declaration of Helsinki. Patients were selected among attendance to the APDP, and sample collection was carried out from April 2007 to August 2009 comprising a total of 240 T1D patients, 167 first-degree relatives (mother and/or father), and 130 unrelated healthy controls living in Portugal. Ninety-seven percent of the patients were Caucasian. Type 1 diabetes diagnosis met the criteria established by the American Diabetes Association. In the family-based collection, the inclusion criteria selected patients with less than 5 years of disease duration.

### Autoantibody analysis

A total of 227 patients and 146 non-affected parents were analyzed for IgM anti-glutamic acid decarboxylase antibodies (IgM anti-GAD) by indirect enzyme-linked immunosorbent assay (ELISA). Briefly, flat-bottomed 96-well ELISA plates were coated overnight at 4 °C, with 1 μg/mL glutamic acid decarboxylase (GAD65/67 C-terminal) peptide (ENZO Life Sciences Inc., Farmingdale, NY, USA) in coating buffer (0.05 M K2PO4). The plates were washed in PBS-Tween, blocked with PBS-BSA 3%, and incubated at 37 °C for 120 min. After washing, four serial dilutions of sera (1:25, 1:50, 1:100, 1:200) were incubated for 180 min at 37 °C and washed. Bound IgM was detected by incubation with 6 μg/mL biotin mouse anti-human IgM antibody (BD Biosciences, Franklin Lakes, NJ, USA) in PBS-Gelatin 1%-Tween 0.075% (1:4000), 4 °C overnight, followed by incubation with streptavidin AKP (1:1000) (Biolegend, San Diego, CA, USA), 37 °C for 2 h, and revealed with the 1 μg/mL pNPP substrate (Sigma-Aldrich, St. Louis, MO, USA). The absorbance at 405 nm was determined using a micro-ELISA plate reader and results were expressed in arbitrary units (AU) calculated as follows: first, sample absorbance was normalized to the absorbance of the serum of a diagnosed T1D patient (positive reference) that was run in all Elisa plates. Second, we corrected this value for inter-plate variation using one sample of a randomly chosen healthy individual (negative reference) that was also run in all the plates. Correction for the variation coefficient of the negative reference was performed according to the following formula: Corrected value = normalized value × (1 − CV), where CV is the coefficient of variation of the negative reference. Patients and relatives were also analyzed for IgG AutoAb seropositivity using commercial standardized clinical laboratory tests for the following antigens: protein tyrosine phosphatase (PTP)-like protein (IA-2 ELISA Version 2 kit, RSR, Cardiff, UK): specificity 99% and sensitivity 63%; glutamic acid decarboxilase (GAD65 Ab ELISA kit, RSR): specificity 98% and sensitivity 92%; and islet cell autoantigens (ICA, Indirect Imunofluorescence, Menarini, Florence, Italy).

### IGH SNP genotyping

Genomic DNA was extracted from whole blood using the Chemagen Magnetic Bead Technology (Chemagic MSM I, Baesweiler, Germany). DNA preparations were quantified using the PicoGreen method (Invitrogen/Life Technologies, Paisley, UK) according to supplier instructions. SNP selection criteria took into account allelic frequency in the Caucasian populations, available sequencing confirmation, and tagging of LD blocks (http://www.hapmap.org). The SNP genotyping method used the Mass Array system to design multiplex reactions for PCR, iPlex primer extension (Sequenom, San Diego, CA, USA) and the MALDI-TOF-based Mass Array platform (Sequenom). A total of 239 patients, 169 non-affected parents, and 130 unrelated healthy controls were genotyped for 15 SNPs mapping in 4 regions in immunoglobulin heavy chain locus: 4 SNPs in the *IgHG*, 4 in *IgHD*, 5 in *IgHM*, and 2 in IgHV regions. Genotyping quality control selected 9 SNPs that yield correct genotyping data according to HapMap control samples and passed the Hardy-Weinberg equilibrium test (*P* > 0.05) with a call rate above 80%.

### Genetic analysis

Analysis of T1D association was performed in two independent datasets. At nominal 0.05 significance level, the case-control dataset has 85% power to detect allelic association effects assuming OR 1.25, 30% allele frequency, and complete LD with trait while the family-based dataset has 74% power to detect allelic transmission disequilibrium assuming OR 2.5, 30% allele frequency, and complete LD with trait. T1D association in the case-control dataset was performed under different genetic models using the PLINK software, and results of the best fitting model are presented (Purcell et al. 2007). Genetic association in the family-based cohort was analyzed with transmission disequilibrium tests (TDT) using the PLINK TDT software. Linkage disequilibrium (LD) analysis was performed using the correlation coefficient (*r* ^2^) to estimate pair-wise LD. The LD map was plotted using the Haploview software to identify LD blocks. Genetic association with IgG AutoAbs was tested using PLINK TDT. Association analysis of anti-GAD IgM titer was performed by quantitative trait loci (QTL) methods implemented in PLINK. Nominal results under the conventional *P* value = 0.05 were considered suggestive evidence for association.

## Results

### Study design

**Fig. 1:**
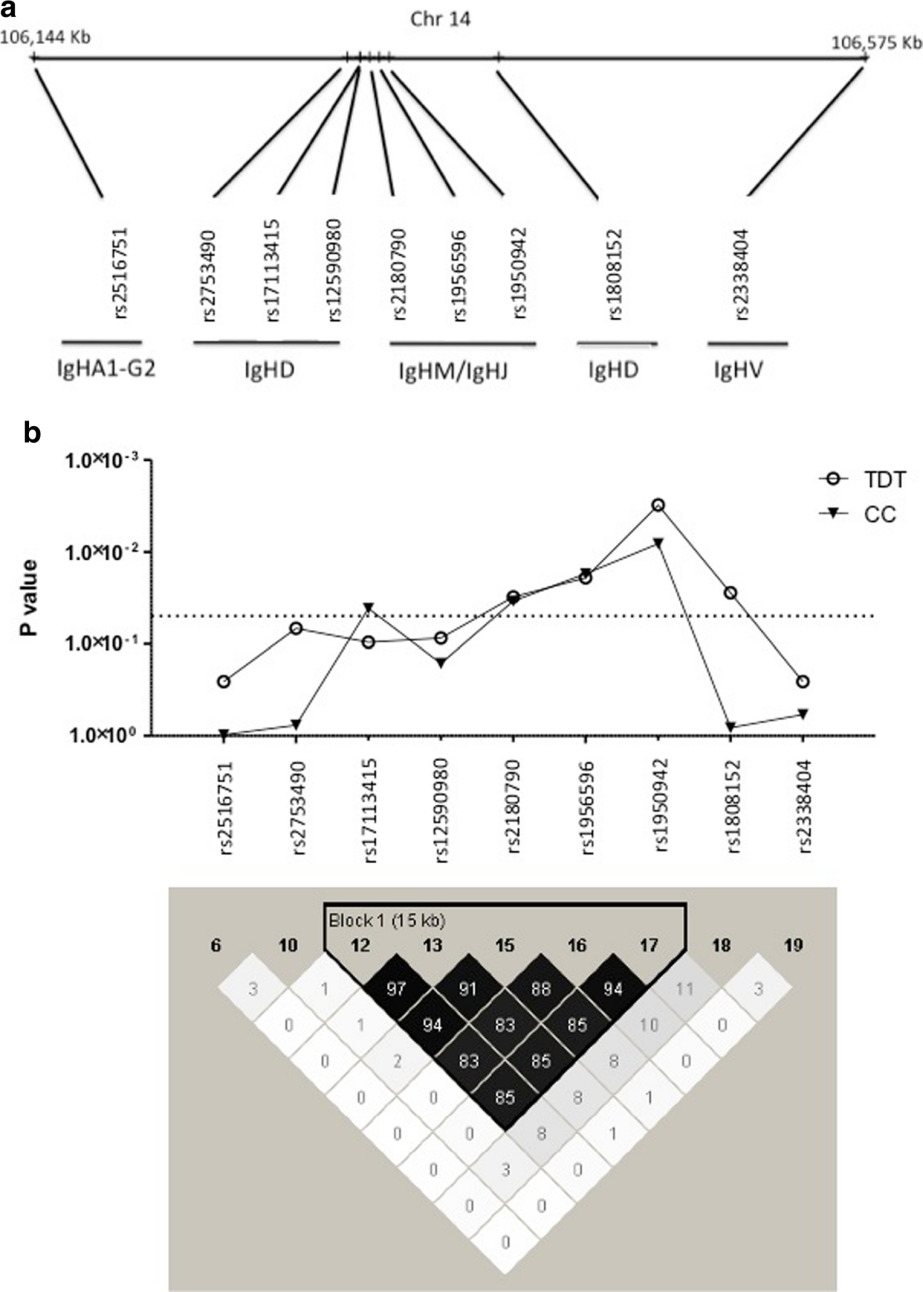
Genetic association of immunoglobulin heavy chain (*IGH*) locus to type 1 diabetes (T1D). **a** Relative physical distances of nine single nucleotide polymorphisms (SNP) across 430 kb in *IGH* locus, in chromosome 14. SNP assignment to *IGH* gene segments is shown (see Table 2 for individual SNP-defined positions). **b** Nominal *P* values of association tests in two cohorts of T1D patients. Case control (*CC*) genotypic analysis under the recessive model was performed in 137 patients and 130 controls and is represented by the *inverted shaded triangles*. Transmission disequilibrium tests (*TDT*) were performed in 102 patients and 169 first-degree relatives and are represented by the *open circles*. *Dashed line* indicates the threshold of suggestive association (*P* = 0.05). Linkage disequilibrium (*LD*) analysis is depicted as a diagram generated by the Haploview software package and values of pair-wise *r*-squared are represented. The *dark shaded area* represents a LD block encompassing five analyzed SNPs in the *IGHD-M* region

We sought to analyze the association of SNPs at the *IGH* locus with T1D and in two patient cohorts. Despite the paucity of markers with common alleles in this region, we identified 9 SNPs spanning 400 kb in the *IGH* locus that passed genotyping quality control criteria (Fig. 1a). In the case-control collection, we compared genotype frequencies of 9 *IgH* SNPs in 137 patients with 130 healthy controls. Using the family-based collection, we performed transmission disequilibrium tests (TDT) in 102 recently diagnosed T1D patients and 169 non-affected parents. *IgH* SNPs showing suggestive association with T1D in either collection were used to test association with auto-reactive antibodies. IgG AutoAb positivity association analysis used TDT in the family-based cohort, while quantitative trait analysis (QTL) of IgM AutoAb titer used either T1D cases of both cohorts and non-affected parents (Table 1). No correlation was found between AutoAb levels at enrollment and age at diagnosis or time since diagnosis (data not shown).

**Table 1:** Study plan and sample size

Phenotype	Type 1 diabetes		IgG anti-GAD	IgG AAbs^e^	IgM anti-GAD
Genetic test	Genot. Assoc.^c^	TDT^d^	TDT ^d^	TDT^d^	QTL^f^
Sample size	137 cases 130 controls	102 cases 169 relatives	57 cases 126 relatives	49 cases 126 relatives	227 cases 146 relatives
Age in cases (years)^a^	31.7/29	19.1/17.4	18.0/17.0	16.5/16	27.3/25.0
Age-at-diagnosis (years)^b^	18.4/18.0 (2–51)	17.4/16.8 (2–28)	15.3/15.8 (2–30)	14.4/15.0 (2–23)	17.4/16.4 (2–47)
Disease duration (years)^a^	13.3/12.0	1.7/1.0	2.6/1.0	1.8/1.0	9.9/7.0
Sex: male patients (%)	56	63	54	59	56

^a^Mean/median

^b^Mean/median (age range)

^c^Genotype association analysis

^d^Transmission disequilibrium test

^e^Multiple autoantibodies

^f^Quantitative trait locus analysis

### IGH locus association with T1D

Case-control genotype association analysis identified four *IGH* SNPs (rs17113415, rs2180790, rs1956596, and rs1950942) showing suggestive association with T1D (*P* < 0.05), which best fitted the recessive model. Three associated SNPs mapped in the *IGHM* gene segment coding for the immunoglobulin M isotype (Fig. 1b, Table 2). Accordingly, transmission disequilibrium analysis in the parent-affected child cohort detected that the same three SNPs in the *IGHM* gene (rs2180790, rs1956596, and rs1950942) were associated with T1D (Fig. 1b, Table 3). In these two independent cohorts, rs1950942 showed the highest association (*P* = 9.35E−03 and *P* = 3.08E−03, respectively), and these concordant results denote that the identified associations are not attributable to population stratification effects. Linkage disequilibrium (LD) analysis revealed a region of strong LD covering 15 kb of the *IGHD* and *IGHM* regions that encompassed the four SNPs associated with T1D, suggesting a single association signal (Fig. 1b). We noted that an additional SNP (rs1808152) mapping 57 kb upstream from this LD block (in the region of *IGH* diversity segments) was over-transmitted to T1D patients (Table 3) and could represent a risk factor in T1D. The coherent results in the two cohorts corroborate that genetic polymorphisms closely mapping in the *IGHM* region show suggestive association with T1D.

**Table 2:** *IGH* SNPs and type 1 diabetes association with the case-control cohort

MARKER	Chr14 (bp)^a^	Gene region	Call rate^b^	Minor allele	MAF^c^	Cases^d^	Controls^d^	OR (95% CI)^e^	*P*
rs2516751	106,143,806	*IGHA1/ IGHG2*	0.87	G	0.48	30/106	21/75	1.01 (0.53–1.90)	9.73E−01
rs2753490	106,306,394	*IGHD*	0.96	T	0.25	8/122	9/118	0.86 0.32–2.30)	7.64E−01
rs17113415	106,312,884	*IGHD*	0.95	T	0.44	22/110	33/88	0.53 (0.29–0.98)	4.11E−02
rs12590980	106,313,201	*IGHD*	0.92	A	0.43	22/114	26/86	0.64 (0.34–1.20)	1.64E−01
rs2180790	106,317,994	*IGHM downst*	0.96	T	0.46	22/109	35/91	0.52 (0.29–0.96)	3.36E−02
rs1956596	106,322,972	*IGHM upst*	0.96	G	0.48	24/105	40/87	0.50 (0.28–0.89)	1.83E−02
rs1950942	106,328,066	*IGHM/ IGHJ6*	0.92	G	0.43	15/103	33/93	0.41 (0.21–0.80)	9.35E−03
rs1808152	106,384,914	*IGHD1–1*	0.88	C	0.34	12/104	14/110	0.91 (0.40–2.05)	8.14E−01
rs2338404	106,575,151	*IGHV*	0.91	A	0.40	19/115	19/95	0.83 (0.41–1.65)	5.88E−01

^a^Positioning in chrom14 (EnsemblGRCh37)

^b^All individuals in the two cohorts

^c^Minor allele frequency

^d^Minor allele homozygous genotypes/alternative genotypes

^e^Odds ratio and 95% confidence interval

**Table 3:** Type 1 diabetes: transmission disequilibrium test for *IGH* SNPs

Marker	MFA^a^	Trans^b^	Untrans^c^	OR^d^	L95^e^	U95^f^	*P*
rs2516751	A	11	17	0.64	0.30	1.38	2.57E−01
rs2753490	T	10	20	0.50	0.23	1.06	6.79E−02
rs17113415	T	13	23	0.56	0.28	1.11	9.56E−02
rs12590980	A	12	22	0.54	0.27	1.10	8.64E−02
rs2180790	T	14	28	0.50	0.26	0.94	3.08E−02
rs1956596	G	13	28	0.46	0.24	0.89	1.92E−02
rs1950942	G	8	25	0.32	0.14	0.71	3.08E−03
rs1808152	C	18	7	2.57	1.07	6.15	2.78E−02
rs2338404	A	17	11	1.54	0.72	3.10	2.57E−01

^a^Minor frequency allele

^b^Minor allele transmission count

^c^Minor allele transmission count

^d^Odds ratio

^e^Lower limit of 95% confidence interval

^f^Upper limit of 95% confidence interval

### IGH region and autoantibody repertoires

**Fig. 2:**
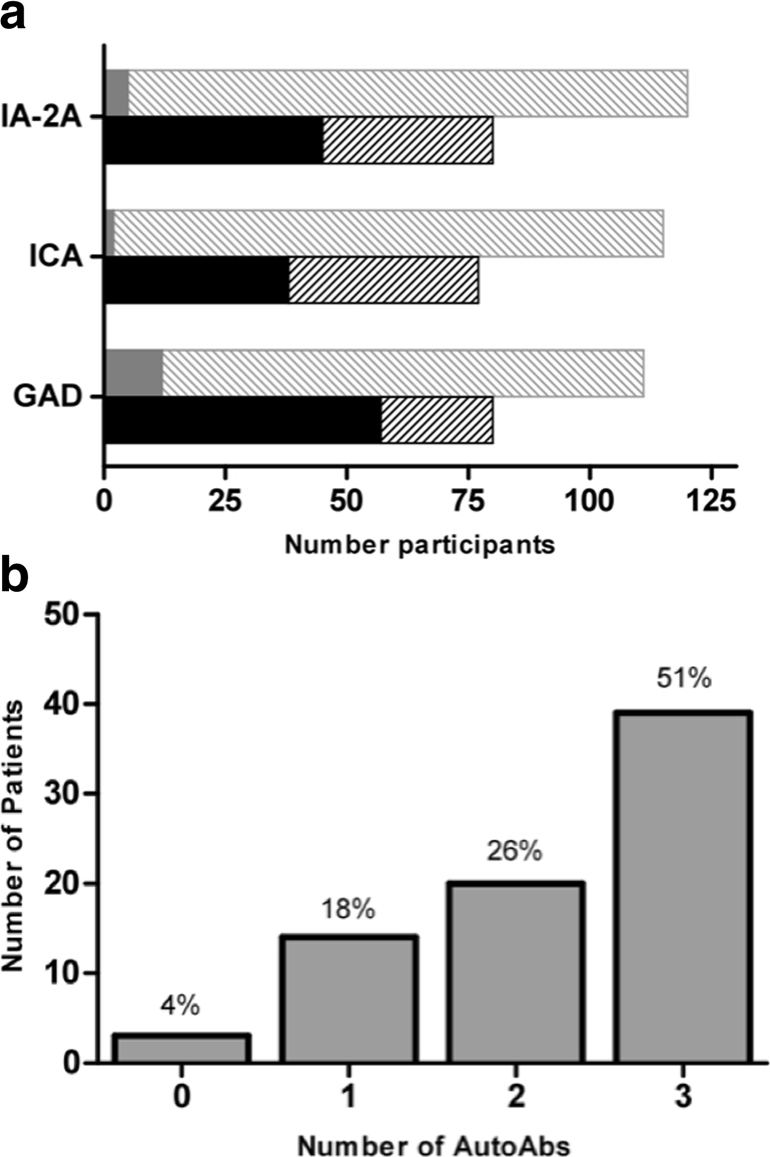
Autoantibodies positivity. **a** Number of T1D patients of the family-based cohort (represented in *black*) or their non-affected parents (represented in *gray*) displaying IgG positivity (*filled area*) or negativity (*striped area*) for the indicated antigens. **b** Number of T1D patients of the family-based cohort showing positivity for the indicated number of antibody specificities against the following antigens: glutamic acid decarboxylase (*GAD*), islet cell autoantigen (*ICA*), and protein tyrosine phosphatase (*PTP*)-like protein (IA-2). Relative frequency of each group is shown as *percent* of the number of patients analyzed

Next, we tested whether the *IGHM* SNPs associated with T1D were also predisposing to antibody positivity in T1D patients. We analyzed 76 patients and 126 unaffected parents of the family-based cohort for three IgG AutoAbs associated with T1D, namely anti-GAD, anti-ICA, and anti-IA-2. The prevalence of these AutoAbs in our patient collection varied from 50 to 75%, while was considerably lower in their unaffected relatives (<11%) (Fig. 2a). AutoAb positivity against each of the three pancreatic-islet antigens was suggestively associated with three SNPs that were also associated with T1D (rs1956596, rs1950942, and rs1808152) (Table 4 and Supp. Tables S2 and S3) suggesting that presence of AutoAbs for multiple antigens could be partially controlled by the *IgH* locus.

**Table 4:** Anti-GAD IgG: transmission disequilibrium test for T1D-associated SNPs in the *IGH* locus

MARKER	MFA^a^	Trans^b^	Untrans^c^	OR^d^	L95^e^	U95^f^	*P*
rs2180790	T	7	13	0.54	0.21	1.35	1.80E−01
rs1956596	G	4	12	0.33	0.11	1.034	4.55E−02
rs1950942	G	3	12	0.25	0.07	0.88	2.01E−02
rs1808152	C	9	2	4.5	0.97	20.8	3.48E−02

^a^Minor frequency allele

^b^Minor allele transmission count

^c^Minor allele non-transmission count

^d^Odds ratio

^e^Lower limit of 95% confidence interval

^f^Upper limit of 95% confidence interval

To determine whether the *IGHM* region was controlling repertoire multi-autoreactivity, we classified patients as mono-autoreactive (if positive for any one AutoAb) or as multi-autoreactive (if positive for two or three AutoAbs). This showed that approximately 77% of the T1D patients were multi-autoreactive (Fig. 2b). Using multi-autoreactivity as affected status, we found that rs1950942 in the *IGHM* region was associated with antibody multi-autoreactivity in T1D patients, as ascertained by TDT (Table 5). Strikingly, this SNP also showed the strongest association with T1D (Fig. 1, Tables 2 and 3), suggesting that its contribution to T1D relates to the generation of multi-autoreactive antibody repertoires.

**Table 5:** Multi-autoreactive IgG: transmission disequilibrium test for T1D-associated SNPs in the *IGH* locus

MARKER	MFA^a^	Trans^b^	Untrans^c^	OR^d^	L95^e^	U95^f^	*P*
rs2180790	T	10	15	0.66	0.30	1.48	3.17E−01
rs1956596	G	7	16	0.43	0.18	1.06	6.06E−02
rs1950942	G	4	14	0.28	0.09	0.87	1.84E−02
rs1808152	C	10	3	3.33	0.91	12.1	5.22E−02

^a^Minor frequency allele

^b^Minor allele transmission count

^c^Minor allele non-transmission count

^d^Odds ratio

^e^Lower limit of 95% confidence interval

^f^Upper limit of 95% confidence interval

**Fig. 3:**
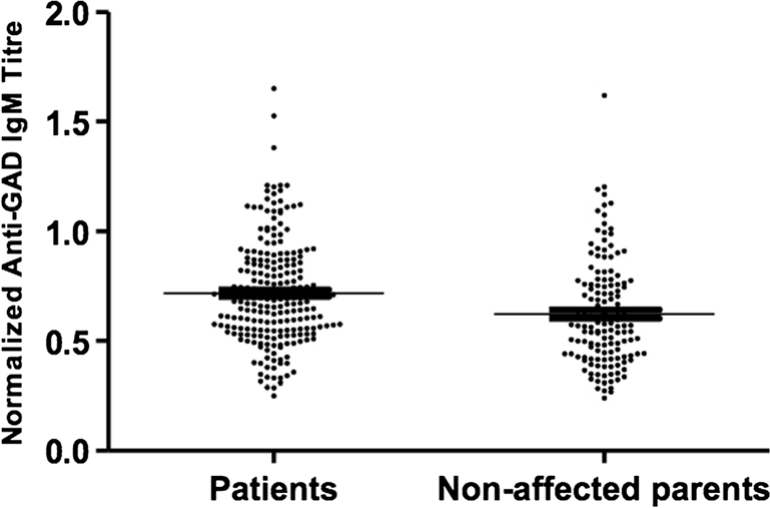
Anti-GAD IgM reactivity. Results from 227 T1D patients and 146 non-affected parents are represented as arbitrary units after normalization, as described in the “Materials and methods” section. *P* < 0.0001, Wilcoxon signed-rank test

**Fig. 4:**
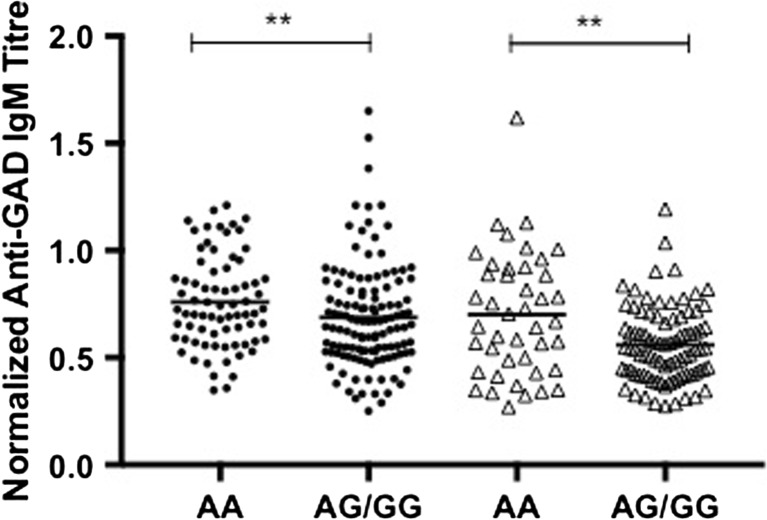
T1D-susceptibility genotype at rs1950942 is associated with increased levels of anti-GAD IgM. Normalized anti-GAD IgM titers in 200 patients (*black circles*) and 129 relatives (*open triangles*) stratified by the T1D-aasocited genotyped at rs1950942 (*AA* versus *AG/GG*). **P < 0.01, Mann-Whitney test (one-tailed)

The location of rs1950942 in the *IGHM* region indicated that this region could control the IgM autoantibody repertoire in T1D patients. Thus, we ascertained serum IgM autoreactivity by measuring anti-GAD reactivity in T1D patients and non-affected parents, with a GAD peptide-based ELISA assay. This anti-GAD ELISA detects low-affinity IgM antibodies, as prior serum incubation with soluble GAD did not impede the detection of anti-GAD reactivity (data not shown). We found that T1D patients had significantly higher levels of anti-GAD reactivity as compared to non-affected parents (Fig. 3). Performing QTL analysis under the additive model in 227 T1D patients, we found that rs1950942 was controlling the levels of anti-GAD IgM reactivity (Table 6). The same SNP was found to control anti-GAD IgM reactivity in non-affected parents (Table 7), suggesting that the *IGH* genetic control of anti-GAD IgM production was not conditioned by the T1D status. This genetic effect is illustrated by the rs1950942 genotype-stratified analysis of anti-GAD IgM levels in patients and relatives (Fig. 4). Together, these data implicate polymorphisms in the *IgM* region of the *IGH* locus in the generation of IgM and IgG AutoAbs in T1D patients and cohesively suggest the involvement of the *IGHM* locus in disease susceptibility.

**Table 6:** Anti-GAD IgM reactivity QTL analysis in T1D patients

MARKER	NMISS^a^	BETA^b^	*r* ^2c^	*P* ^d^
rs2180790	216	−0.021	0.010	1.39E−01
rs1956596	213	−0.027	0.016	6.56E−02
rs1950942	200	−0.042	0.037	5.98E−03
rs1808152	191	−0.004	0.0004	7.90E−01

^a^Number of non-missing genotypes

^b^Regression coefficient

^c^Regression *r*-squared

^d^Wald test *P* value

**Table 7:** Anti-GAD IgM reactivity QTL analysis in patient parents

Marker	NMISS^a^	Beta^b^	*r* ^2c^	*P* ^d^
rs2180790	139	−0.038	0.028	4.88E−02
rs1956596	140	−0.039	0.030	3.92E−02
rs1950942	129	−0.057	0.062	4.17E−03
rs1808152	114	0.021	0.007	3.71E−01

^a^Number of non-missing genotypes

^b^Regression coefficient

^c^Regression *r*-squared

^d^Wald test *P* value

## Discussion

We report that polymorphisms in the *IGHM* gene region show suggestive association with T1D development and with AutoAb repertoires. Nevertheless, studies on genetic association of AutoAbs in T1D that used data from genome-wide SNP scans did not identify the *IGH* locus (Plagnol et al. 2011). We reason that current human genome variation databases incorporate an unusual scarcity of consensus SNPs at the *IGH* locus. Consequently, the *IGH* locus and in particular the *IGH* constant region, determining the Ig isotype class, are poorly represented in the SNP arrays commonly used in GWAs studies (Supp. Table S1). We have hand-picked 15 SNPs spanning approximately 400 kb from the *IGHA* gene segment to the V region in the *IGH* locus, but only 9 SNPs were successfully genotyped. This possibly reflects expected difficulties in obtaining SNPs with high allele call rate at this locus, which undergoes somatic genetic rearrangements represented in peripheral blood B cells.

The analysis of two independent T1D Portuguese cohorts replicated the association of T1D development with three SNPs mapping in the *IGHM* gene region (coding for the IgM isotype). Linkage disequilibrium analysis revealed that these three SNPs were encompassed within a 15-kb region of strong LD block together with one SNP in the *IGHD* gene (IgD isotype) that showed marginal T1D association in the case-control cohort. Although these cohorts have relatively small sizes, these cohesive findings are suggestive of genetic association of the *IGHM* region with T1D. Nevertheless, the reported results warrant studies in larger cohorts to test replication of such T1D association signals in other populations. It is worth mentioning that despite very low *IGH* coverage, one genome-wide scan found that rs1981496 (mapping within the *IGHV* region and 250 kb upstream of LD block here reported) was associated with T1D at a significance level of *P* = 9.95E−05 (Burton et al. 2007). Although imputation analysis on GWAS data was not feasible due to scarcity of SNPs in this region, this observation corroborates the possibility that the *IGH* locus encloses association signals to T1D susceptibility.

Interestingly, the T1D association results highlighted rs1950942 in a region close to an *IGH* somatic recombination site at the boundary of the *IGHM* and *IGH*J regions. As *IGH* recombination sites are major determinants of antibody specificity, our results point to a link of *IGH* variants with the generation of AutoAb repertories in T1D. Genetic determination of self-reactivity should be detectable in primary repertoires, represented by the IgM low-affinity antibodies that do not result from antigen-driven affinity maturation occurring during adaptive immune responses. It has been demonstrated that anti-islet IgM reactivity is precociously detected in T1D (Hoppu et al. 2004b). In particular, one study reported increased concentration of circulating serum IgM in recently diagnosed T1D patients accompanied by increased IgM and also IgG binding to fixed rat islet cells (Decraene et al. 1992). Another study showed that positivity for both GAD and IA-2 antibodies at birth was associated with a 7.5-fold increased risk for developing T1D in a cohort of Danish children (Eising et al. 2011). We found that the levels of IgM with anti-GAD reactivity were significantly higher in T1D patients when compared to their parents. Strikingly, analysis of the genetic control of IgM anti-GAD reactivity in T1D patients and non-affected parents by the *IGH* locus converged in rs1950942, which maps in the *IGHM* region and was also the most associated with T1D. These results support the hypothesis that *IGH* variants contribute to the genetic control of natural IgM self-reactivity. We speculate that deviations of the natural IgM reactivity could fuel chronic pancreatic auto-reactivity that generates IgG AutoAbs accompanying progression to T1D. Consistent with this hypothesis, rs1950942 in the *IGHM* was associated with positivity to IgG AutoAbs and with IgG autoantibody multi-reactive repertoire in T1D patients.

A GWAS remarked that the majority of T1D regions did not associate with autoantibody positivity (Plagnol et al. 2011). Also, HLA alleles associated with AutoAbs in T1D patients were shown to be distinct from T1D susceptibility conferred by the HLA locus (Howson et al. 2011). These results suggest that known T1D susceptibility loci were dissociated from the genetic determination of IgG AutoAbs in T1D patients.

Overall, our results suggest that the *IGH* genetic variation is associated with T1D susceptibility by shaping IgM reactivity and subsequently predisposing to the generation of IgG AutoAbs in the course of the T1D natural history. Confirmation of our proposal that the *IGH* locus takes part in the genetic component of T1D susceptibility warrants replication of these findings in larger patient collections.

## Electronic supplementary material

ESM 1 (PDF 282 kb)
