# Supplementary material for: Immunoglobulin M gene association with autoantibody reactivity and type 1 diabetes
Source: Immunogenetics. 2017 May 22;69(7):429–37. doi: 10.1007/s00251-017-0999-1 (PMC5486809; doi:10.1007/s00251-017-0999-1)
Supplement: Supplementary file 1 — (PDF 282 kb) [file 251_2017_999_MOESM1_ESM.pdf]

Immunoglobulin M gene association with autoantibody reactivity and Type 1 Diabetes

by

Inês Rolim [1,2], Nádia Duarte [1], Gabriela Barata [2,3], João Costa [1], Luís Gardete-Correia [3], José Boavida [3], Rui Duarte [3], João Raposo [3], Zulmira Peerally [3], Manuela Catarino [2], Carlos Penha-Gonçalves[1,3].

Affiliations:

[1] Instituto Gulbenkian de Ciência, Oeiras, Portugal

[2] Faculdade de Farmácia, Universidade de Lisboa, Lisboa, Portugal

[3] Portuguese Diabetes Association, Education and Research Center, Lisboa, Portugal

Corresponding Author:

Carlos Penha-Gonçalves

Instituto Gulbenkian de Ciência, Apartado 14, P-2781-901 Oeiras, Portugal

Phone: +351 21 4464614

Fax: +351 21 4407970

e-mail: cpenha@igc.gulbenkian.pt

Supplementary information Contents:

Table S1……………………………………………………………………………………………..2

Table S2……………………………………………………………………………………………..3

Table S3……………………………………………………………………………………………..4

Table S1. SNPs in genome-wide genotyping platforms mapping in the IgH region (106.0 MB-106.7Mb, Ensembl release GRh37.p10).

Illumina 550K		*Affymetrix 500K*	
SNP ID	*Position in Chrom 14*	*SNP ID*	*Position Chrom 14*
rs8003220	106,072,316	rs10147883	106,098,346
rs2516780	106,137,467	rs2753509	106,328,827
rs7494172	106,175,202	rs41351146	106,475,997
rs11628672	106,199,579	rs1988091	106,614,665
		rs1981496	106,645,846
		rs8012888	106,675,615
		rs2073678	106,758,084
		rs8005585	106,927,499
		rs2467919	106,964,357
		rs12885703	106,972,767
		rs17114279	106,990,000

Table S2. Anti-ICA IgG positivity: Transmission Disequilibrium Test for T1D-associated SNPs in the IGH locus.

MARKER	*Minor Allele*	*Trans*[(a)]	*Untrans*[(b)]	*OR*[(c)]	*L95*[(d)]	*U95*[(e)]	*P*
rs2180790	T	4	9	0.44	0.13	1.923	1,66E-01
rs1956596	G	2	9	0.22	0.04	4.455	3.48E-02
rs1950942	G	1	8	0.12	0.01	5.444	1.96E-02
rs1808152	C	7	3	2.33	0.60	1.6	2.06E-01

a, minor allele transmissions count; b, minor allele non-transmissions count; c, odds ratio; d, Lower limit of 95% confidence interval; e, Upper limit of 95% confidence interval.

Table S3. Anti-IA2A IgG positivity: Transmission Disequilibrium Test for T1D-associated SNPs in the IGH locus.

MARKER	*Minor Allele*	*Trans*[(a)]	*Untrans*[(b)]	*OR*[(c)]	*L95*[(d)]	*U95*[(e)]	*P*
rs2180790	A	5	17	0.29	0.10	0.7972	1,05E-02
rs1956596	T	5	19	0.26	0.09	0.7048	4,27E-03
rs1950942	T	3	15	0.2	0.05	0.6908	4,68E-03
rs1808152	C	9	3	3	0.81	11.08	8,33E-02

a, minor allele transmissions count; b, minor allele non-transmissions count; c, odds ratio; d, Lower limit of 95% confidence interval; e, Upper limit of 95% confidence interval.
